# Partial purification and characterization of an antimicrobial activity from the wood extract of mangrove plant Ceriops decandra

**DOI:** 10.17179/excli2015-741

**Published:** 2016-02-09

**Authors:** Aritra Simlai, Kalishankar Mukherjee, Anurup Mandal, Kashinath Bhattacharya, Amalesh Samanta, Amit Roy

**Affiliations:** 1 Department of Biotechnology, Visva-Bharati University, Santiniketan, West Bengal, India; 2 Department of Chemistry, Visva-Bharati University, Santiniketan, West Bengal, India; 3 Division of Microbiology, Department of Pharmaceutical Technology, Jadavpur University, Kolkata, India; 4 Department of Botany, Visva-Bharati University, Santiniketan, West Bengal, India

**Keywords:** antimicrobial activity, bioactivity guided fractionation, Ceriops decandra, diterpenoids, human pathogens, mangroves

## Abstract

The development of resistance towards the antibiotics in use today has been a source of growing concern in the modern healthcare system around the world. To counter this major threat, there is an urgent need for discovery of new antimicrobials. Many plants, like mangroves, possess highly diversified list of natural phytochemicals which are known to have wide range of bioactivities. These phytochemicals can be good sources for the discovery of new drugs. In this study, we report the partial phytochemical characterization and antimicrobial activities of a semi-purified fraction isolated from the wood tissue of *Ceriops decandra*, a mangrove plant. This fraction named CD-3PM was chromatographically separated from *C. decandra* wood extract and was subjected to different spectral analyses to determine its partial chemical nature. The structural investigation indicates the presence of two diterpenoids, i) 3β, 13β-Dihydroxy-8-abietaen-7-one and ii) 3β-Hydroxy-8,13-abietadien-7-one in the CD-3PM fraction. The antimicrobial potential of this fraction was evaluated by microdilution-MTT assay against several organisms. Among the nine microorganisms found to be sensitive to the CD-3PM fraction, six organisms are reported to be pathogenic in nature. The CD-3PM fraction with broad spectrum antimicrobial efficacy revealed the presence of two diterpenoids and possesses potential applications in drug discovery process and food processing industries.

## Introduction

Antimicrobial properties reported earlier in the tissue extracts of *Ceriops decandra* (Vadlapudi and Naidu, 2009[27]; Chandrasekaran et al., 2009[4]; Ravikumar et al., 2010[19]; Simlai and Roy, 2012[24]), have led us to investigate the partial structural nature and growth inhibitory potential of a semi-purified phytochemical fraction from this plant. The fraction named CD-3PM has been isolated from the wood tissue of this plant using chromatographic methods. *Ceriops decandra* (Griff.) Ding Hou is a shrubby mangrove species of the Rhizophoraceae family found in the Sundarban estuary in India. The species has been reported to be used by traditional healers as relief for wounds, boils, hepatitis, ulcers, angina, diabetes, diarrhea and dysentery (Watt and Breyer-Brandwijk, 1962[31]; Duke and Wain, 1981[5]; Kathiresan and Ramanathan, 1997[9]; Bandaranayake, 1998[1]; Simlai and Roy, 2013[25]). Previously a number of phytochemicals have been isolated and identified from *C. decandra *(Wu et al., 2008[33]; Wang et al., 2012[30]; Nebula et al., 2013[14]), but these compounds have not been linked with the antimicrobial activities reported from the crude tissue extracts of this species (Vadlapudi and Naidu, 2009[27]; Chandrasekaran et al., 2009[4]; Ravikumar et al., 2010[19]; Simlai and Roy, 2012[24]). Vundru et al. (2013[28]) revealed the presence of a wide range of phytochemicals as indicated by gas chromatography-mass spectrometry (GC-MS) analysis in *C. decandra* crude leaf extracts with antifungal and larvicidal properties. A study by Sakagami et al. (1998[20]) is the only report in which the authors used fractionated leaf extracts of the plant containing lignin-like polyphenolic structures demonstrating its protective effect in mice infected with lethal dose of *E. coli*. Apart from the observations made by Sakagami et al. (1998[20]), we have not come across any other scientific report that assigns the observed antimicrobial activities seen in this plant to one or more phytochemical components in a pure or semi-pure state (Wu et al., 2008[33]; Wang et al., 2012[30]; Nebula et al., 2013[14]). In this study, we have described the isolation and partial chemical characterization of a substantially purified phytochemical fraction named CD-3PM from the wood tissue of *C. decandra*. The present study also shows growth inhibitory potential of the fraction CD-3PM on some microbes, some of which are traditionally considered as threats to human health. 

## Materials and Methods

### General experimental procedures

The extracted antimicrobial fraction, CD-3PM, obtained from preparative thin layer chromatography (TLC) plates (described below) was analyzed by doing high performance liquid chromatography (HPLC) (Prominence, Shimadzu) using a C-18 column (Phenomenex Luna, 4.6 × 250 mm, 5 µm) at a flow rate of 1 mL/min with solvents acetonitrile and water. The semi-purified fraction was subjected to GC-MS analysis on a gas chromatograph (Trace GC Ultra, Thermo Scientific) hyphenated to mass spectrometer (Polaris Q, Thermo Scientific). The sample was injected into a Thermo TR-WaxMS column (30 m × 0.25 mm, 0.25 µm) and helium gas (99.999 %) at a flow rate of 1 mL/min was used as the carrier gas. For MS detection, the ion source temperature was maintained at 230 °C, electron ionization (EI) was performed with an ionization energy of 70 eV and mass range at *m/z* 40 - 600. The Fourier transform infrared (FTIR) analysis was carried out on a Spectrum 100 FTIR spectrometer (Perkin Elmer) by taking 1 mg of the sample in thin film of potassium bromide (KBr). The FTIR spectra were recorded within the range of 450 - 4000 cm^-1^. Nuclear magnetic resonance (NMR) data for both ^1^H and ^13^C spectra were recorded on a Bruker spectrometer (400 MHz) using deuterated chloroform (CDCl_3_) as the solvent.

### Plant materials

*Ceriops decandra *(Griff.) Ding Hou wood samples were collected from Sundarban estuary, West Bengal. The dried specimen of this plant after proper identification was deposited at the Herbarium of Botany department, Visva-Bharati University (Accession no. VB/BH/01861). The wood from the plant was washed thoroughly under tap water and then with distilled water and air-dried in shade for several weeks. The tissue were then ground into fine powder using an electric grinder and stored at room temperature until use.

### Extraction and bioactivity guided isolation of CD-3PM fraction

Powdered wood (1.35 kg) of *C. decandra* was sequentially extracted with hexane, benzene and chloroform in increasing order of polarity for approximately 46 h using a 5 L soxhlet apparatus. The temperature for extractions was 72 °C for hexane and benzene and 61 °C for chloroform. The extracts obtained were filtered using Whatman No. 1 filter paper. The filtered extracts were then concentrated under reduced pressure using a rotary evaporator and left at room temperature for drying. 

These extracts were tested for their antimicrobial activities using disc diffusion assay (Bauer et al., 1966[3]) as described previously (Simlai and Roy, 2012[24]). The benzene extract which seemed to indicate maximum antimicrobial activity, was subjected to further separation using preparative TLC method in the next step. For most preparative TLC runs, the plates were prepared using Silica gel 60 PF_254_ containing gypsum (Merck) as the matrix. BEA [benzene: ethanol: ammonia (18:2:0.2)] was used as the mobile phase. After the separation, the TLC plates were allowed to dry at room temperature so that no residue of the mobile phase remained in the plate. One of these preparative TLC chromatograms was used for activity-guided bioautography (Gupta et al., 2010[8]) in an attempt to identify the major antibacterial fraction position on the TLC plates. Using this bioautogram as reference, the silica containing antimicrobial fraction was scraped off from the untreated TLC plate. This silica was then extracted with chloroform to obtain the major antimicrobial fraction and concentrated under reduced pressure. This entire process was repeated several times until a good amount of the initial antimicrobial fraction was obtained. In the final step of purification, a concentrated solution of this initial fraction was run again on a high resolution commercial preparative TLC plates [PLC silica gel 60 F_254+366_, 2 mm (Merck)] using BEA as mobile phase. The area containing antimicrobial activity was similarly identified on this plate, scraped and extracted with chloroform. The sample was dried and re-extracted once with methanol. This methanol fraction was termed CD-3PM and air-dried and stored at 4 °C until use.

### Structural characterization

Partial structural characterization of the CD-3PM fraction was carried out using analytical techniques like GC-MS, FTIR, ^1^H and ^13^C NMR as described above. 

### Test microorganisms 

The evaluation of the antimicrobial activity in this study was performed using seven Gram-positive bacterial strains viz., *Bacillus subtilis* (MTCC 121), *Bacillus coagulans*, *Bacillus cereus *(ATCC 11778), *Bacillus polymyxa* (NCTC 4747), *Bacillus pumilus *(ATCC 14884), *Micrococcus luteus* (ATCC 10240), *Staphylococcus aureus *(ATCC 29737); eight Gram-negative bacterial strains viz., *Shigella sonnei *(NK 4010), *Klebsiella pneumoniae *(ATCC 10031),* Bordetella bronchiseptica *(ATCC 4617),* Pseudomonas aeruginosa *(ATCC 25619),* Providencia *spp., *Salmonella choleraesuis *(NCTC 36), *Salmonella typhimurium *(NCTC 74), *Salmonella *F 14669 and four fungal strains *Saccharomyces* *cerevisiae*, *Candida albicans*, *Microsporum gypseum*, *Cryptococcus *spp*.* For the last three fungal strains, spores were used for the antimicrobial assay.

### Antimicrobial activity evaluation

The antimicrobial potential of CD-3PM fraction was checked by determining its minimum inhibitory concentration (MIC) for the test organisms. The assay was performed by microdilution method using 96-well flat-bottom micro-test plates in sterile environment as described by Malekinejad et al. (2011[12]) with minor modifications. Briefly, 5×10^4^ number of CFU (bacteria and *S. cerevisiae*) or fungal spores in each well were incubated in 100 µL fresh culture media containing CD-3PM fractions of different concentrations obtained by serial dilution (1:1) technique. The plates in sealed boxes were incubated at respective temperatures for 8 h (bacteria) to 42 h (fungus). After the incubation, 20 µL of 3-(4,5-dimethyl-2thiazolyl)-2,5-diphenyl tetrazolium bromide (MTT) solution (5 mg/mL in PBS) was added into each well and incubated at respective temperature for 30 min and 2 h for bacteria and fungi, respectively. The formazan crystals formed were dissolved adding 100 µL of acidic isopropanol (36 mM HCl in isopropanol) into each well and was read at 570 nm with 630 nm as the reference filter using a micro plate reader (SpectraMax M3, Molecular devices) (Mosmann, 1983[13]). The concentration at which the absorbance was equal or lower than that of its corresponding control (well received no bacteria) was considered to be the MIC value. Each test was performed in duplicate using appropriate controls. Ampicillin and fluconazole were used as standards. Dimethylsulfoxide (DMSO), the CD-3PM/antibiotic solubilizer served as the negative control.

## Results and Discussion

### Extraction and isolation of CD-3PM fraction

In our earlier study, it has been reported that the chloroform extract of wood from *C. decandra* possesses considerable antibacterial activity (Simlai and Roy, 2012[24]). Although several other tissues of *C. decandra* exhibited broad spectrum activities, their extracts failed to exhibit such activities when separated by TLC (Simlai and Roy, 2012[24]). The wood tissue of *C. decandra* has been considered for further study with respect to the isolation and identification of the antimicrobial activity, because the extracts from this source appeared to retain their bioactivities even after the TLC procedure.

The hexane, benzene and chloroform sequential extracts of *C. decandra* wood tissues obtained by soxhlet extraction have been analyzed for their antibacterial activity using disc diffusion assay against *B. subtilis* and *B. coagulans*. The results are represented in Table 1(Tab. 1) which indicates clearly that the wood tissue benzene extract exhibits the maximum activity. Therefore, the benzene extract has been considered for isolation and partial characterization of the antimicrobial component(s). The chloroform extract also has exhibited good amount of antimicrobial activities. Analysis of the benzene and chloroform extracts by using bioautography seems to indicate they essentially contain the same major components (AS & AR Unpublished observations). 

**Figure 1:**
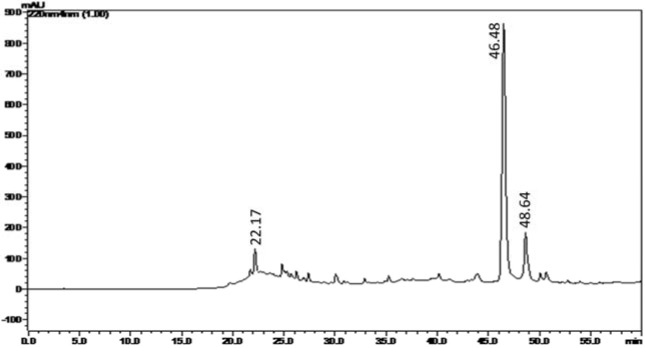
Analytical HPLC (C-18, 5 µm, 4.6 × 250 mm) chromatogram of CD-3PM fraction isolated from the wood of *C. decandra*. Spectral data was recorded at 220 nm.

In the next step, the benzene extract of *C. decandra* wood has been subjected to preparative TLC in order to identify and collect the bioactive fraction/s. For identifying the bioactive zone on the TLC bed, the reference TLC plate has been bioautographed using *B. subtilis*. The bioautogram after MTT assay has been found to exhibit the inhibition zone along *R_f_* 0.577. After determination of location of the antimicrobial activity in the reference bioautogram, the silica in the corresponding area of the experimental TLC plate has been scraped off and extracted using chloroform to obtain the initial antimicrobial fraction. This initial fraction has been subjected to one more round of similar purification on commercial preparative TLC plate (Merck) to obtain the antimicrobial fraction termed CD-3PM as described in the 'Materials and Methods' section. When analyzed by HPLC method (Figure 1(Fig. 1)), the CD-3PM fraction has indicated the presence of a large peak at retention time 46.48 min and two smaller ones at 22.17 min and 48.64 min.

### Partial structural characterization of CD-3PM fraction 

The partial structural characterization of the CD-3PM fraction indicates the presence of two major compounds (compound 1 and compound 2) in this fraction. To identify the organic functional groups, infrared (IR) spectroscopy of the CD-3PM fraction has been carried out and the FTIR spectral data is shown in Table 2(Tab. 2). The analysis revealed that the spectral pattern of the two compounds 1 and 2 are almost similar to each other. The signals in the IR spectra at 3410 and 3415 cm^-1^ indicate the presence of hydroxyl (-OH) group in both the compounds 1 and 2. The absorptions at 1727 and 1730 cm^-1^ are suggestive of the presence of keto (C=O) group in the compounds. The appearance of the bands at 1690 and 1645 cm^-1^ can be attributed to unsaturation present in both the compounds. 

The fraction CD-3PM has been subjected to GC-MS analysis and the mass spectra obtained in the procedure by electron ionization technique helped to interpret the molecular formula of the two compounds in the fraction. The molecular formula for compound 1 has been assigned as C_20_H_32_O_3_ exhibiting molecular ion peak at *m/z* 320. Compound 2 has been assigned the molecular formula C_20_H_30_O_2 _based on EI-MS molecular ion peak at *m/z* 302. Other significant ion peaks appeared in the mass spectrum of the compound 1 are, *m/z* 302 (M-18), 284 (M-18-18), 234 (M-86), while in the mass spectrum of the compound 2, prominent peaks observed besides molecular ion peak at *m/z* 284.

The ^1^H and ^13^C NMR studies of the CD-3PM fraction in deuterated chloroform (CDCl_3_) have been carried out at 400 MHz and 100 MHz, respectively. The chemical shift values pertaining to ^1^H NMR analysis along with their probable assignments with respect to compounds 1 and 2 are represented in Table 3(Tab. 3). The interpretation of the spectral data suggests the presence of very minor difference with regard to the proton environment of both the molecules which is otherwise identical. The chemical shifts indicated by signals around δ 0.83, 1.20, 1.30 in the compound 1 and at δ 0.84, 1.21 and 1.33 in the compound 2 suggest the presence of five C-CH_3_ groups in both of these compounds. The appearance of one proton signal at δ 3.15 in both the compounds indicate the presence of CHOH linkage while the appearance of two protons signals at δ 2.40 and 2.45 respectively in the compounds 1 and 2 are suggestive of the presence of keto methylene (-CH_2_CO) grouping. The presence of an olefinic proton in compound 2 is indicated by the appearance of signal at δ 5.30. The ^13^C NMR data as represented in Table 4(Tab. 4) have been found to be similar for both the compounds except the appearance of two additional peaks at δ 114.1 and δ 115 for unsaturated (sp^2^) carbon atoms in the compound 2.

**Figure 2:**
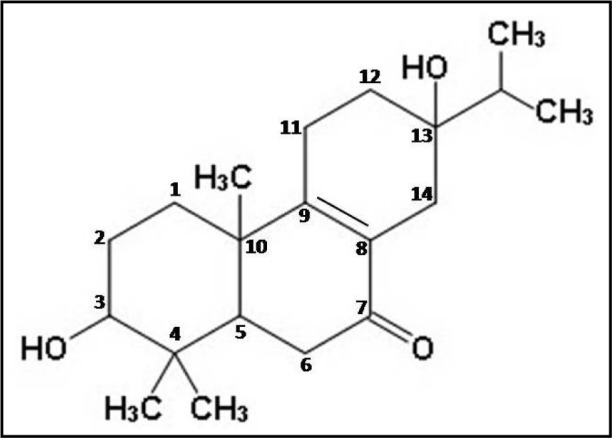
The probable structure of compound 1 (C_20_H_32_O_3_)

**Figure 3:**
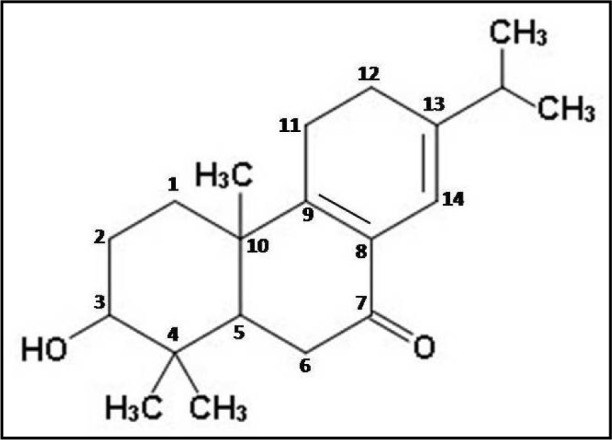
The probable structure of compound 2 (C_20_H_30_O_2_)

Thus, the different spectral analyses of the antimicrobial fraction CD-3PM have revealed the presence of two diterpenoid type of natural compounds in this fraction and their most probable structures may be represented respectively as 3β,13β-Dihydroxy-8-abietaen-7-one for compound 1 (Figure 2(Fig. 2)) and 3β-Hydroxy-8,13-abietadien-7-one for compound 2 (Figure 3(Fig. 3)). It is to be noted that Wang et al. (2013[29]) described the presence of 11 diterpenes from *C. decandra* and the above mentioned compounds 1 and 2 have similarities with Decandrin B and Decandrin H respectively, reported in that communication. However, no report on the biological activity such as antimicrobial activities has been made with respect to Decandrin B and H. Probably due to close resemblances in the structural properties of the two diterpenoid compounds 1 and 2, it was not possible to cleanly separate the two compounds from each other, even after repeated trials. As a result it is not possible to decide whether the observed antimicrobial activities of CD-3PM described in the next section, are due to one or the other of the two compounds or whether they both have antimicrobial activities.

The minor differences between compounds 1 and 2 with that of Decandrins B and H respectively, may be attributed to differential environmental factors on *C. decandra* plants growing in two different geographical locations. The plant samples used in this study were collected from Sundarban areas in the east coast of India whereas in case of the study by Wang et al. (2013[29]), the samples were collected from Godavari estuary in the south-east coast of India. The reason behind the difference in synthesis of these compounds by the plant may be due to the differences in microenvironments and biotic-abiotic stress factors in two geographically distinct coastal areas. This is in line with the earlier observation of Gupta et al. (2014[7]) that

“i) environmental factors dictate the secondary metabolites profiles of medicinal plants and

ii) the same plant species growing in different natural habitats around the world may have different metabolite profiles with respect to these compounds.”

### Antimicrobial activities of CD-3PM fraction

Determination of antimicrobial activities by MIC evaluation using microdilution-MTT assay has been carried out by reading the intensity of the purple coloured formazan crystals formed on reduction of the yellow dye MTT. The reduction occurs due to mitochondrial dehydrogenase of the living cells and is consequently directly proportional to the viable bacterial/fungal density (Malekinejad et al., 2011[12]; Shi et al., 2008[23]). The antimicrobial fraction CD-3PM has been found to demonstrate promising broad spectrum activity when evaluated using a number of pathogenic strains including Gram-positive, Gram-negative bacteria and fungi and is represented in Table 5(Tab. 5). The CD-3PM fraction has exhibited potential MIC values against the Gram-positive bacteria *B. subtilis* (62.5 µg/mL), *B. coagulans *(62.5 µg/mL), *B. cereus *(125 µg/mL), *B. polymyxa* (15.62 µg/mL), *B. pumilus *(125 µg/mL), *M. luteus* (125 µg/mL), Gram-negative bacteria* S. sonnei *(125 µg/mL), *K. pneumoniae* (500 µg/mL) and the fungus *S. cerevisiae* (78.12 µg/mL). Rest of the organisms tested have been found to be resistant to the maximum concentration of the CD-3PM fraction used in the assay.

The variation in sensitivity to antibiotics, external agents and detergents between Gram-positive and Gram-negative bacteria have already been reported earlier where Gram-negatives have been found to exhibit more resistance to these external agents. This can be attributed to the presence of lipopolysaccharides in their outer membranes, which make these cells impermeable to many of these drugs (Nikaido and Vaara, 1985[15]) whereas the Gram-positive bacteria possess an outer peptidoglycan layer, which is an inefficient permeability barrier (Scherrer and Gerhardt, 1971[21]) thus making the Gram-positives much more vulnerable to external agents, such as drugs, compared to Gram-negatives. Among the nine microorganisms found to be sensitive to the CD-3PM fraction, six organisms have been reported to be pathogenic in nature. *B. subtilis* usually non-pathogenic, has been reported to cause food poisoning and infections in immuno-compromised patients (Logan, 1988[11]). *B. cereus* has been known to cause food borne illness, ocular and skin infections (Kotirantaa et al., 2000[10]), *B. pumilus* to be the causative agent in cases of cutaneous infections, food poisoning (Tena et al., 2007[26]; From et al., 2007[6]) and *M. luteus* in cases of intracranial abscesses, pneumonia, septic arthritis, meningitis and endocarditis in humans (Bannerman and Peacock, 2007[2]). The Gram-negative bacteria *S. sonnei* has been reported to cause shigellosis, a food borne dysenteric disease (Niyogi, 2005[16]) whereas* K. pneumoniae *causes nosocomial infections like pneumonia, meningitis etc. (Podschun and Ullmann, 1998[17]). A recent surveillance on antimicrobial resistance by World Health Organization (WHO) reported *Shigella* species to have developed resistance to fluoroquinolones and *K. pneumoniae *to third generation cephalosporins and carbapenems (WHO, 2014[32]). Particular concern has been expressed on the development of resistance by *K. pneumoniae* to carbapenems, generally the last line of treatment available (WHO, 2014[32]). 

A recent study estimated that at least 50,000 deaths occur each year across Europe and the US alone due to infections by antimicrobial-resistant organisms with many hundreds of thousands more deaths going unreported in other parts of the globe (RAR, 2014[18]). The study predicts premature deaths of 300 million people due to drug resistance over the next 35 years. Therefore, in order to control this burgeoning menace, along with other measures, there's a pressing need for development of newer antimicrobials not based on the existing synthetic antibiotics (Shah, 2005[22]). A continuous search for the development of new antimicrobials must be carried out and plants due to their diversified nature of secondary metabolites have proved to be a valuable source for this purpose. The current study reveals the promising antimicrobial potential of a partially purified fraction isolated from the wood tissue of *Ceriops decandra*, a mangrove plant. This fraction CD-3PM has exhibited significant broad-spectrum growth inhibitory activities against a number of organisms, many of which are considered as human pathogens. Partial characterization of this antimicrobial fraction indicates the presence of two diterpenoids in the fraction, whose most probable structures have been determined as 3β,13β-Dihydroxy-8-abietaen-7-one (compound 1) and 3β-Hydroxy-8,13-abietadien-7-one (compound 2). The findings from this study suggest that the CD-3PM fraction possesses effective antimicrobial activities and must be studied further to unveil its potential for application in pharmaceutical as well as food processing industries.

## Acknowledgements

This work has been supported by a grant from the University Grants Commission [F. No. 40-176/2011 (SR)] to AR. Authors wish to profusely thank the Director, Bose Institute and Prof. Parimal Chandra Sen for the use of institutional HPLC, GC-MS, FTIR facilities and Mr. Swaroop Biswas, Mr. Smriti Ranjan Maji, Mr. Pranab Dey for assistance with the HPLC, GC-MS, FTIR experiments there, Dr. Kartick Chandra Bhowmick, Department of Chemistry, Visva-Bharati for ^1^H and ^13^C NMR experiments and UPE-II programme (UGC), Jadavpur University for providing some experimental facilities. Authors also wish to thank the Director, IICB, Kolkata, for initial *C. decandra* samples and Dr. Kumudranjan Naskar for plant identification.

## Conflict of interest

The authors declare that there are no conflicts of interest.

## Figures and Tables

**Table 1:**
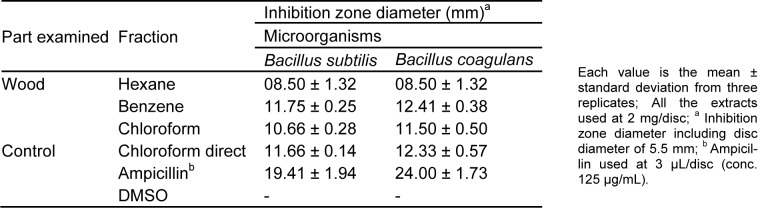
Comparative antibacterial activity of *C. decandra *wood tissue extracts obtained during soxhlet extraction by disc diffusion assay

**Table 2:**
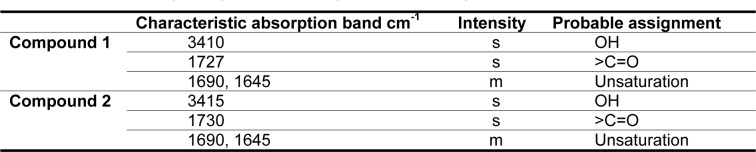
Infrared absorption spectrum of compound 1 and compound 2

**Table 3:**
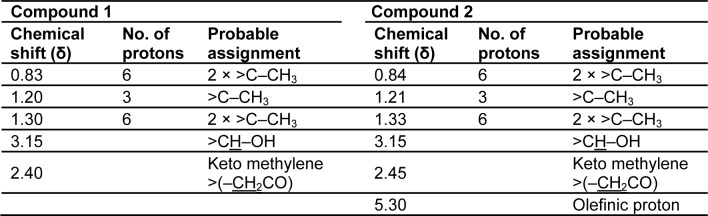
^1^H NMR proton signals of compound 1 and compound 2 in CDCl_3_ at 400 MHz

**Table 4:**
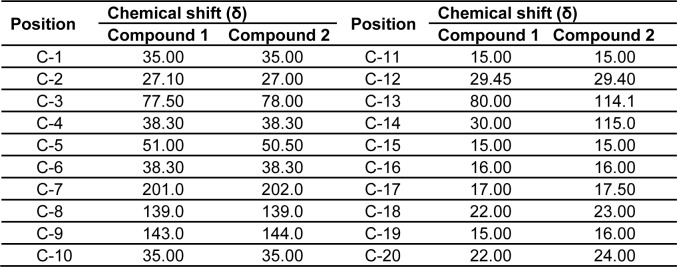
^13^C NMR data of compound 1 and compound 2 in CDCl_3_ at 100 MHz

**Table 5:**
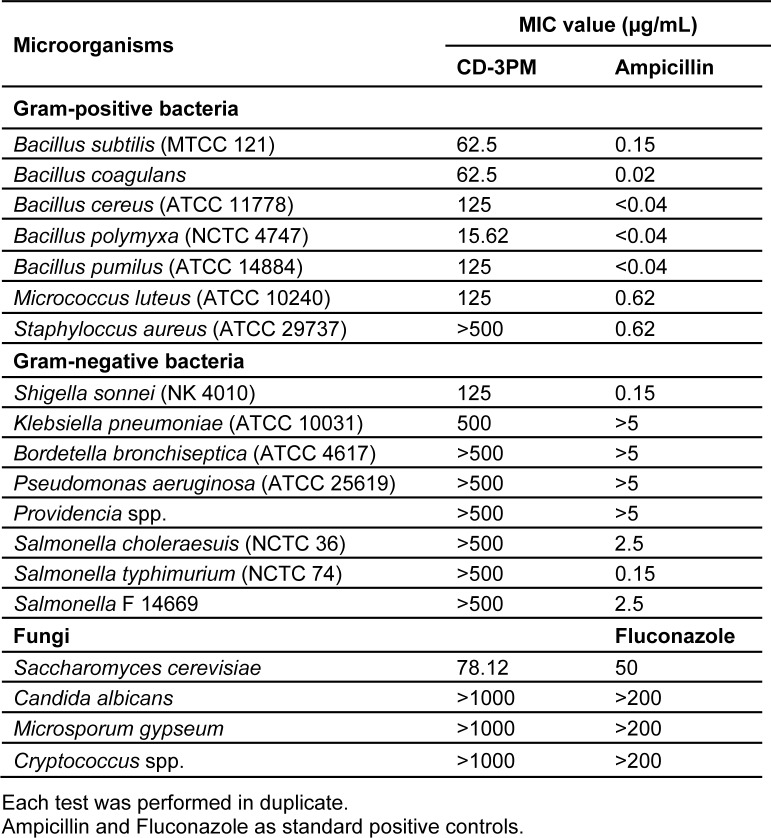
MIC value determination of CD-3PM fraction by microdilution assay
